# Supplementary material for: SARS-CoV-2 m-RNA Vaccine Response in Immunocompromised Patients: A Monocentric Study Comparing Cancer, People Living with HIV, Hematopoietic Stem Cell Transplant Patients and Lung Transplant Recipients
Source: Vaccines (Basel). 2023 Jul 26;11(8):1284. doi: 10.3390/vaccines11081284 (PMC10459936; doi:10.3390/vaccines11081284)
Supplement: Supplementary file 1 [file vaccines-11-01284-s001.zip › vaccines-2460937-supplementary.pdf]

SUPPLEMENTARY DATA

Supplementary Table S1: Characteristics of cancer patients

**Variables**	**Cancer (n=232)**
Solid tumor	
yes	196 (84%)
no	36 (16%)
Cancer stage	
0	1 (1%)
1	5 (4%)
2	9 (7%)
3	10 (8%)
4	106 (81%)
missing values	101
**Cancer treatment**	
Chemotherapy	
yes	83 (36%)
Alkylating agents	57 (25%)
Antimetabolites	44 (19%)
Anti-tumor antibiotics	10 (4%)
Topoisomerase inhibitors	21 (9%)
Mitotic inhibitors	26 (11%)
no	149 (64%)
Surgery	
yes	32 (14%)
no	200 (86%)
Radiation	
yes	48 (21%)
no	184 (79%)
Hormonal therapy	
yes	30 (13%)
no	202 (87%)
Immunotherapy	
yes	37 (16%)
no	195 (84%)
Corticosteroids/target therapy	
yes	53 (23%)
no	179 (77%)
Follow-up	
yes	67 (29%)
no	165 (71%)

Supplementary Table S2: Characteristics of PLWH

**variables**	**PLWH (n=131)**
Antiretroviral therapy	
yes	131 (100%)
CDC stage	
A	81 (63%)
B	25 (20%)
C	22 (17%)
missing values	3
CD4-T cell (mean; SD)	646.9 (±294.5)
CD4-T cell (%) (mean; SD)	34.2 (±10.9)
HIV-1 RNA result	
undetectable, ≤ 20 copies/ml	108 (84%)
detectable, > 20 copies/ml	20 (16%)
missing values	3
HIV-1 RNA (mean; SD)	42.3 (±33.7)
missing values	111

PLWH: people living with HIV

Supplementary Table S3: Characteristics of HSCT patients

Variables	HSCT (n=126)
Months between transplant and vaccine (median; Q1-3)	15.31 (6.7 - 27.3)
Active GVHD	
Acute GvHD	2 (2%)
Chronic GvHD	35 (28%)
No active GvHD	89 (71%)
Active malignancies	
yes	8 (6%)
no	118 (94%)
CD4-T cell (median; Q1-3)	226 (111 - 423)
missing values	13
**Immunosuppressive drugs**	
Cyclosporine	
yes	6 (5%)
no	120 (95%)
Prednisone ≥ 10mg/d	
yes	11 (9%)
no	115 (91%)
Tacrolimus	
yes	14 (11%)
no	112 (89%)
MMF	
yes	2 (2%)
no	124 (98%)
Ruxolitinib	
yes	16 (13%)
no	110 (87%)
**Current oncologic treatment**	
Sorafenib	
no	126 (100%)
Midostaurine	
no	126 (100%)
5azacytidine	
yes	6 (5%)
no	120 (95%)
Venetoclax	
yes	4 (3%)
no	122 (97%)
other	
yes	1 (1%)
no	125 (99%)
Rituximab < 6 months	
yes	5 (4%)
no	121 (96%)

HSCT: hematopoietic stem cell transplantation

Supplementary Table S4: Lung transplant patients

Variables	Lung transplant (n=39)
Months between lung transplant and vaccine (median; Q1-3)	81.6 (28.5 - 120.6)
**Etiology of lung disease before transplant**	
Bronchiectasis	3 (8%)
COPD	18 (46%)
Cystic fibrosis	7 (18%)
Interstitial lung disease	9 (23%)
Pulmonary hypertension	2 (5%)
Cortico Steroid Inhalation (CSI)	
yes	2 (5%)
no	37 (95%)
Non-Invasive Ventilation (NIV)	
yes	1 (3%)
no	38 (97%)
O2 received	
yes	1 (3%)
no	38 (97%)
High dose steroids during the last 12 months	
Yes	11 (28%)
No	28 (72%)
Triple immunosuppression	
Yes	33 (85%)
No	6 (15%)
**Ongoing immunosuppressive treatments**	
Prednisone	
yes	34 (87%)
no	5 (13%)
Dosage of Prednisone (median; Q1-3)	5 (5 - 5)
missing values	5
Tacrolimus	
yes	35 (90%)
no	4 (10%)
Dosage of Tracrolimus (median; Q1-3)	4 (2.5 - 6)
missing values	4
Everolimus	
yes	4 (10%)
no	35 (90%)
Dosage of Everolimus (median; Q1-3)	2.25 (1.4 - 3.5)
missing values	35
Azathioprine	
yes	1 (3%)
no	38 (97%)
Mycophenolate mofetil	
yes	37 (95%)
no	2 (5%)
Dosage of Mycophenolate (median; Q1-3)	1440 (780 - 2000)
missing values	2
